# Likelihood-free simulation-based optimal design with an application to spatial extremes

**DOI:** 10.1007/s00477-015-1067-8

**Published:** 2015-04-12

**Authors:** Markus Hainy, Werner G. Müller, Helga Wagner

**Affiliations:** Department of Applied Statistics, Johannes Kepler University, Altenberger Strasse 69, 4040 Linz, Austria

**Keywords:** Simulation-based optimal design, Approximate Bayesian computation, Importance sampling, Spatial extremes, Max-stable processes

## Abstract

In this paper we employ a novel method to find the optimal design for problems where the likelihood is not available analytically, but simulation from the likelihood is feasible. To approximate the expected utility we make use of approximate Bayesian computation methods. We detail the approach for a model on spatial extremes, where the goal is to find the optimal design for efficiently estimating the parameters determining the dependence structure. The method is applied to determine the optimal design of weather stations for modeling maximum annual summer temperatures.

**Electronic supplementary material:**

The online version of this article (doi:10.1007/s00477-015-1067-8) contains supplementary material, which is available to authorized users.

## Introduction

Collecting spatial data efficiently (see eg. Müller 2007) is a problem that is frequently neglected in applied research, although there is growing literature on the subject. Various spatial sampling and monitoring situations such diverse as e.g. for stream networks (Dobbie et al. 2008), water (Harris et al. 2014) and air quality (Bayraktar and Turalioglu 2005), soil properties (Lesch 2005 and Spöck and Pilz 2010), radioactivity (Melles et al. 2011), biodiversity (Stein and Ettema 2003), or greenland coverage (Mateu and Müller 2012) are discussed therein.

Those approaches predominately follow a (parametric) model-based viewpoint. Here, the inverse of the Fisher information matrix represents the uncertainties involved and it is its minimization through a prudent choice of monitoring sites that is desired. This corresponds to the selection of inputs or settings (the design) in an experiment and can thus draw from the rich literature on optimal experimental design (see eg. Fedorov 1972 or Atkinson et al. 2007). There a so-called design criterion, usually a scalar function of the information matrix, is optimized by employing various algebraic and algorithmic techniques. Often the design criterion can be interpreted as an expected utility of the experiment outcome (the collected data), and if this expected utility is an easy to evaluate function of the design settings, the optimal design can be found analytically. In Bayesian design, the design criterion is usually some measure of the expected information gain of the experiment (see e.g. Hainy et al. 2014), which is also called the expected utility. As utility function one would typically use convex functionals of the posterior distribution, such as the Kullback-Leibler divergence between the (uninformative) prior and the posterior distribution, to measure the additional information gained by conducting the experiment (Chaloner and Verdinelli 1995).

For problems where neither maximization of the design criterion nor the integration to evaluate the expected utility can be performed, simulation-based techniques for optimal design were proposed in Müller (1999) and Müller et al. (2004). For instance, the expected utility can be approximated by Monte Carlo integration over the utility values with respect to the prior predictive distribution.

In Bayesian design problems, the utility is typically a complex functional of the posterior distribution. Hence, a strategy could be to generate values for the parameters by employing simulation methods like Markov chain Monte Carlo (MCMC) and use these to approximate the utility values. However, as one has to generate a sample from a different posterior for each utility evaluation, this can be computationally very expensive.

We will further assume that the likelihood is not available analytically. In that case it is not possible to employ standard Bayesian estimation techniques. Therefore, we propose to use approximate Bayesian computation (ABC) methods for posterior inference. It is our new approach to utilize these methods for solving optimal design problems. We will also present a solution to quickly re-evaluate the utility values for different posterior distributions by using a large pre-simulated sample from the model.

We illustrate the application of the methodology to derive optimal designs for spatial extremes models. As noted in Erhardt and Smith (2012), models specifically designed for extremes are better suited than standard spatial models to model dependence for environmental extreme events such as hurricanes, floods, droughts or heat waves. A recent overview of modeling approaches for spatial extremes data is given in Davison et al. (2012). We will focus on models for spatial extremes based on max-stable processes to derive optimal designs for the parameters characterizing spatial dependence.

Max-stable processes are useful for modeling spatial extremes as they can be characterized by spectral representations, where spatial dependence can be incorporated conveniently. A drawback of max-stable processes is that closed forms for the likelihood function are typically available only for the bivariate marginal densities. Hence, inference using ABC as in Erhardt and Smith (2012) is a natural avenue. Often the so-called Schlather model (Schlather 2002) is employed, which models the spatial dependence in terms of an unobserved Gaussian process. It usually creates a more realistic pattern of spatial dependence than the deterministic shapes engendered by the so-called Smith model (Smith 1990), which is another very popular model for spatial extremes. Moreover, simulations from the Schlather model can be obtained fairly quickly compared to more complex models, which is important when using a simulation-heavy estimation technique such as ABC.

In our application we consider optimal design for the parameters characterizing the dependence structure of maximum annual summer temperatures in the Midwest region of the United States of America. The problem is inspired by the work of Erhardt and Smith (2014), who use data from 39 sites to derive a model for pricing weather derivatives. Our aim is to rank those sites with respect to the information they provide on the unknown dependence parameters. In this the paper is comparable to Chang et al. (2007), who employ a different entropy-based technique in a similar context. Note, however, that our approach is not limited to this specific application, but could be easily adapted for other purposes.

Shortly before finalizing a first technical report on this topic (Hainy et al. 2013a), we have learned of the then unpublished paper by Drovandi and Pettitt (2013), wherein similar ideas have been developed independently. However, while the basic concept of fusing a simulation-based method with ABC is essentially the same, our approach differs in various ways, particularly on how the posterior for the utility function is generated. Furthermore, we additionally suggest ways of how the methodology can be turned sequential so as to be made useful for adaptive design situations. A very general version of our concept is introduced in Hainy et al. (2013b), whereas in the current exposition we give a detailed explanation of how to employ it in a specific practical situation.

The paper is structured as follows. Section 2 reviews the essentials of simulation-based optimal design as well as the various improvements and modifications lately suggested. Sect. 3 is the core of the paper and details our approach to likelihood-free optimal design with a brief section on essentials of approximate Bayesian computation. Section 4 provides an overview of modeling spatial extremes based on max-stable processes. These are needed in the application in Sect. 5. Finally, Sect. 6 provides a discussion and gives some directions for future research.

The programs for the application were mainly written in R. The R-programs include calls to compiled C-code for the computer-intensive sampling and criterion calculation procedures. We used and adapted routines from the R-packages evd (Stephenson 2002) and SpatialExtremes (Ribatet and Singleton 2013) to analyze and manipulate the data and to simulate from the spatial extremes model. For simulating large samples or performing independent computations, we used the parallel computing and random number generation functionalities of the R-packages snow (Tierney et al. 2013) and rlecuyer (Sevcikova and Rossini 2012). All the computer-intensive parallelizable operations were conducted on an SGI Altix 4700 symmetric multiprocessing (SMP) system with 256 Intel Itanium cores (1.6 GHz) and 1 TB of global shared memory.

## Simulation-based optimal design

We consider an experiment where output values (observations) ${\mathbf{z}}\in {{\fancyscript{Z}}}$ are taken at input values constituting a design ${\varvec{\xi}}$. A model for these data is described by a likelihood $p_{\varvec{\xi}}({\mathbf{z}}|{\varvec{\vartheta}})$, where ${\varvec{\vartheta}}\in \Theta$ denotes the model parameters.

Optimal design, see eg. Atkinson et al. (2007), generally has the goal to determine the optimal configuration ${\varvec{\xi}}^*$ with respect to a criterion $U({\varvec{\xi}})$,

$${\varvec{\xi}}^* = \arg \underset{\varvec{\xi}}{\sup} \,U({\varvec{\xi}}), \quad {\varvec{\xi}}\in \Xi.$$

We adopt a Bayesian approach and assume that a prior distribution $p({\varvec{\vartheta}})$ is specified to account for parameter uncertainty. The prior distribution usually does not depend on the design ${\varvec{\xi}}$. If $u({\mathbf{z}},{\varvec{\vartheta}},{\varvec{\xi}})$ denotes a utility function and $p_{\varvec{\xi}} ({\mathbf{z}},{\varvec{\vartheta}}) = p_{\varvec{\xi}} ({\mathbf{z}}|{\varvec{\vartheta}}) p({\varvec{\vartheta}})$ is the joint density of ${\mathbf{z}}$ and ${\varvec{\vartheta}}$, the expected utility is given as

2.1 $$U({\varvec{\xi}}) = \int _{{\varvec{\vartheta}}\in \Theta} \int _{{\mathbf{z}}\in {\fancyscript{Z}}} u({\mathbf{z}},{\varvec{\xi}},{\varvec{\vartheta}}) \; p_{\varvec{\xi}} ({\mathbf{z}},{\varvec{\vartheta}}) \; {\mathrm{d}} {\mathbf{z}}\; {\mathrm{d}} {\varvec{\vartheta}}.$$

For reasonable choices of utility functions and a detailed introduction into Bayesian optimal design see Chaloner and Verdinelli (1995).

In many applications, neither analytic nor numerical integration is feasible, but simulation-based design can be performed by approximating the criterion by Monte Carlo integration,

$$U({\varvec{\xi}}) \approx \hat{U}({\varvec{\xi}}) = \frac{1}{K} \sum _{k=1}^K u({\mathbf{z}}^{(k)},{\varvec{\xi}},{\varvec{\vartheta}}^{(k)}),$$

if samples $\{({\mathbf{z}}^{(k)},{\varvec{\vartheta}}^{(k)}), \, k=1,\ldots ,K \}$ from the joint distribution $p_{\varvec{\xi}}({\mathbf{z}},{\varvec{\vartheta}})$ can be generated and the utility $u(.)$ is easy to evaluate. Sampling from the joint distribution can typically be performed by sampling ${\varvec{\vartheta}}$ from its prior distribution and ${\mathbf{z}}$ from the likelihood $p_{\varvec{\xi}}({\mathbf{z}}|{\varvec{\vartheta}})$.

Often however, design criteria are not straightforward to evaluate as they require some integration: classical criteria, e.g. based on the Fisher information matrix such as D-optimality, are defined as expected values of some functional with respect to the likelihood, $p_{\varvec{\xi}}({\mathbf{z}}|{\varvec{\vartheta}})$ (Atkinson et al. 2007), whereas Bayesian utility functions, e.g. the popular Kullback-Leibler divergence/Shannon information, are expected values with respect to the posterior distribution of the parameters, $p_{\varvec{\xi}}({\varvec{\vartheta}}|{\mathbf{z}})$ (Chaloner and Verdinelli 1995). Thus, we can write $u({\mathbf{z}},{\varvec{\xi}},{\varvec{\vartheta}})=u({\mathbf{z}},{\varvec{\xi}})$, since the parameters ${\varvec{\vartheta}}$ are integrated out in a Bayesian utility function.

If $\hat{u}({\mathbf{z}},{\varvec{\xi}})$ denotes an approximation of the utility, $U({\varvec{\xi}})$ can be approximated by

2.2 $$ \hat{U}({\varvec{\xi}})= \frac{1}{K} \sum _ {k=1}^K \hat{u}({\mathbf{z}}^{(k)},{\varvec{\xi}}), $$

where ${\mathbf{z}}^{(k)}$ is sampled from the prior predictive distribution $p_{\varvec{\xi}}({\mathbf{z}})$. We will focus on this case in the rest of the paper.

A very general form of simulation-based design, which was proposed by Müller (1999), further fuses the approximation and the optimization of $U({\varvec{\xi}})$ and could be employed here as well. However, for simplicity in this paper we consider only cases with finite design space $\Xi$, where ${\text {card}}(\Xi )$ is small and thus it is feasible to compute $U({\varvec{\xi}})$ for each value ${\varvec{\xi}}\in \Xi$ and rank the results.

We further assume that neither the likelihood nor the posterior is available in closed form. Hence we will use ABC methods to sample from the posterior distribution to approximate the Bayesian design criterion, see Sect. 3 for a detailed description.

We will also consider the more general case where the prior distribution of the parameters, $p({\varvec{\vartheta}})$, is replaced by the posterior distribution, $p_{{\varvec{\xi}}_0}({\varvec{\vartheta}}|{\mathbf{z}}_0)$, which depends on observations ${\mathbf{z}}_0$ previously collected at design points ${\varvec{\xi}}_0$. Thus, information from these data about the parameter distribution can be easily incorporated into the approximation of the utility.

## Likelihood-free optimal design

In this section we will elaborate on particular aspects of simulation-based optimal design without using likelihoods. The general concept was introduced in Hainy et al. (2013b) and termed “ABCD” (approximate Bayesian computation design). The first two subsections review some basic notions of ABC, whereas the last presents two variants for approximating a design criterion by $\hat{U}({\varvec{\xi}})$. This can eventually be optimized to yield

$$ {\varvec{\xi}}^* \simeq \arg \underset{\varvec{\xi}}{\sup} \, \hat{U}({\varvec{\xi}}), \quad {\varvec{\xi}}\in \Xi $$

by stochastic optimization routines (see Huan and Marzouk 2013), which are designed to deal with noisy objective functions, or—as in our example—various designs can be directly compared with respect to their approximated criterion value $\hat{U}$.

### Approximate Bayesian computation (ABC)

To tackle problems where the likelihood function cannot be evaluated, likelihood-free methods, also known as approximate Bayesian computation, have been developed. These methods have been successfully employed in biogenetics (Beaumont et al. 2002), Markov process models (Toni et al. 2009), models for extremes (Bortot et al. 2007), and many other applications, see Sisson and Fan (2011) for further examples.

ABC methods rely on sampling ${\varvec{\vartheta}}$ from the prior and auxiliary data ${\mathbf{z}}^{*}$ from the likelihood to obtain a sample from an approximation to the posterior distribution $p_{\varvec{\xi}}({\varvec{\vartheta}}|{\mathbf{z}})$. This approximation is constituted from draws for ${\varvec{\vartheta}}$ where ${\mathbf{z}}^{*}$ is in some sense close to the observed ${\mathbf{z}}$.

More formally, let $d({\mathbf{z}},{\mathbf{z}}^{*})$ be a discrepancy function that compares the observed and the auxiliary data (cf. Drovandi and Pettitt 2013). In most cases, $d({\mathbf{z}},{\mathbf{z}}^{*}) = d_s({\mathbf{s}}({\mathbf{z}}),{\mathbf{s}}({\mathbf{z}}^{*}))$ for a discrepancy function $d_s(.,.)$ defined on the space of a lower-dimensional summary statistic ${\mathbf{s}}(.).$ An ABC rejection sampler iterates the following steps:

This algorithm draws from the ABC posterior

3.1 $$ \tilde{p}_{\varvec{\xi}}({\varvec{\vartheta}}|{\mathbf{z}}) \propto p({\varvec{\vartheta}}) \int _{{\mathbf{z}}^{*}\in {\mathcal{Z}}} K_{\epsilon}(d({\mathbf{z}},{\mathbf{z}}^{*})) \; p_{\varvec{\xi}}({\mathbf{z}}^{*}|{\varvec{\vartheta}}) \; {\mathrm{d}} {\mathbf{z}}^{*}, $$

where $K_{\varepsilon}(d) = (1/\varepsilon ) K(d/\varepsilon )$ is the uniform kernel with bandwidth ${\varepsilon}$, i.e. $K_{\varepsilon}(d({\mathbf{z}},{\mathbf{z}}^{*})) \propto {\mathbb{I}}(d({\mathbf{z}},{\mathbf{z}}^{*}) \le {\varepsilon} )$. Here ${\mathbb{I}}(d({\mathbf{z}},{\mathbf{z}}^{*}) \le {\varepsilon} )$ is the indicator function which takes the value 1 if $d({\mathbf{z}},{\mathbf{z}}^{*}) \le \varepsilon$ and 0 otherwise. The ABC posterior $\tilde{p}_{\varvec{\xi}}({\varvec{\vartheta}}|{\mathbf{z}})$ is equal to the targeted posterior $p_{\varvec{\xi}}({\varvec{\vartheta}}|{\mathbf{z}})$ if the summary statistic ${\mathbf{s}}({\mathbf{z}})$ is sufficient and $K_{\varepsilon}(d({\mathbf{z}},{\mathbf{z}}^{*}))$ is a point mass at the point ${\mathbf{z}}^{*}={\mathbf{z}}$.

If $K_{\varepsilon}(d)$ is a more general smoothing kernel, e.g. the Gaussian or the Epanechnikov kernel, the resulting ABC posterior can be sampled using importance sampling (cf. e.g. Fearnhead and Prangle 2012). Let $q({\varvec{\vartheta}})$ denote a proposal density for ${\varvec{\vartheta}}$ with sufficient support (at least the support of $p({\varvec{\vartheta}})$), then ABC importance sampling can be performed as follows:

As the likelihood terms $p_{\varvec{\xi}}({\mathbf{z}}^{*}_r|{\varvec{\vartheta}}_r)$ cancel out in the weights, explicit evaluation of the likelihood function is not necessary.

Algorithm 2 produces a weighted approximation $\{({\varvec{\vartheta}}_r,{\mathbf{z}}^{*}_r),W_r\}_{r=1}^R$ of the augmented distribution

3.3 $$ \tilde{p}_{\varvec{\xi}}({\varvec{\vartheta}},{\mathbf{z}}^{*}|{\mathbf{z}}) \propto K_{\varepsilon}(d({\mathbf{z}},{\mathbf{z}}^{*})) \; p_{\varvec{\xi}}({\mathbf{z}}^{*}|{\varvec{\vartheta}}) \; p({\varvec{\vartheta}}) $$

and hence the marginal sample $\{{\varvec{\vartheta}}_r,W_r\}_{r=1}^R$ is an approximation of the marginal ABC posterior given in Eq.  (3.1). Obviously, the ABC rejection sampler is a special case of the importance sampler, where the proposal distribution is the prior, i.e. $q({\varvec{\vartheta}}) = p({\varvec{\vartheta}})$, and the non-normalized importance weights are either equal to zero or one.

### Accuracy of ABC

ABC estimates suffer from different sources of approximation error: first, choosing the tolerance level ${\varepsilon} >0$ has the consequence that only an approximation to the targeted posterior is sampled. Second, even for ${\varepsilon} \rightarrow 0$ the sampled distribution $\tilde{p}({\varvec{\vartheta}}|{\mathbf{z}})$ does not converge to the (true) posterior distribution if the summary statistic is not sufficient. Finally, sampling introduces a Monte Carlo error, which depends on sampling efficiency and sampling effort. Sampling efficiency is measured by the *effective sample size (ESS)*, which is the number of independent draws required to obtain a parameter estimate with the same precision (see Liu 2001).

The tolerance level ${\varepsilon}$ plays an important role as it has an impact on the quality of the ABC posterior $\tilde{p}_{\varvec{\xi}}({\varvec{\vartheta}}|{\mathbf{z}})$ as an approximation to the target posterior $p_{\varvec{\xi}}({\varvec{\vartheta}}|{\mathbf{z}})$ as well as on the effective sample size. For ABC rejection sampling, the effective sample size is equal to the number of accepted draws. Reducing ${\varepsilon}$ leads to an increase of the rejection rate, and hence the sampling effort in order to maintain a desired ESS will be higher.

For importance sampling the ESS is given as

$$ {\text{ESS}}= \frac{R}{1 + {\text{CV}}(w)}, $$

where ${\text{CV}}(w)$ denotes the coefficient of variation of the importance weights (see Liu 2001). It can be estimated by

3.4 $$ \widehat{\text{ESS}} = \frac{\left( \sum _{r=1}^R w_r \right) ^2}{\sum _{r=1}^R \left( w_r\right) ^2}= \frac{1}{\sum _{r=1}^R \left( W_r\right) ^2}.$$

As more imbalanced weights result in a lower effective sample size, the choice of ${\varepsilon}$ directly affects the ${\text{ESS}}$ of the importance sample. Weights become more imbalanced with decreasing tolerance level ${\varepsilon}$, see Eq. (3.2), resulting in a lower ${\text{ESS}}$. Consider e.g. $q({\varvec{\vartheta}}) = p({\varvec{\vartheta}})$, where the importance weights are $W_r \propto K_{\varepsilon}(d({\mathbf{z}},{\mathbf{z}}^{*}_r))$. For ${\varepsilon} \rightarrow \infty$, weights are constant, $W_r \propto 1$, and hence the ${\text{ESS}}$ takes its maximal value $R$, whereas for ${\varepsilon} \rightarrow 0$, many weights will be close to or equal to zero. Therefore, there is a trade-off between closeness of the ABC posterior to the true posterior, which is achieved by choosing ${\varepsilon}$ as small as possible, and a close to optimal effective sample size.

### Utility function estimation using ABC methods

We consider Bayesian information criteria, where the utility function, $u({\mathbf{z}},{\varvec{\xi}})$, is a functional of the posterior distribution, $p_{\varvec{\xi}}({\varvec{\vartheta}}|{\mathbf{z}})$. Based on information-theoretic grounds, a widely used utility function is the Kullback-Leibler (KL) divergence between the prior and the posterior distribution (see Chaloner and Verdinelli (1995) and the references given therein). Precise estimation of the KL divergence is difficult and requires large samples from the posterior distribution (for an estimation approach see Liepe et al. 2013). However, if the posterior distribution has a regular shape, i.e., if it is unimodal and does not exhibit extreme skewness and kurtosis as in our example, then the posterior precision is also a good measure of the posterior information gain (see also Drovandi and Pettitt 2013). The posterior precision utility defined as

$$ u({\mathbf{z}},{\varvec{\xi}}) = 1/\det \left( {\text {Var}}_{\varvec{\xi}}({\varvec{\vartheta}}|{\mathbf{z}}) \right) $$

can be efficiently estimated from the sample variance-covariance matrix. We will use it in our example in Sect. 5.

For an intractable likelihood, a sample obtained by ABC methods can be used to approximate the utility function $u({\mathbf{z}},{\varvec{\xi}})$ by $\hat{u}_{LF}({\mathbf{z}},{\varvec{\xi}})$. The expected utility Eq. (2.1) at design point ${\varvec{\xi}}$ can then be approximated by

$$ {\hat{U}}({\varvec{\xi}}) = \frac{1}{K} \sum _{k=1}^K \hat{u}_{LF}({\mathbf{z}}^{(k)},{\varvec{\xi}}). $$

The sample $Z = \{{\mathbf{z}}^{(k)}\}_{k=1}^K$ from the prior predictive distribution $p_{\varvec{\xi}}({\mathbf{z}})$ can be generated by first drawing ${\varvec{\vartheta}}^{(k)} \sim p({\varvec{\vartheta}})$ and then ${\mathbf{z}}^{(k)} \sim p_{\varvec{\xi}}({\mathbf{z}}|{\varvec{\vartheta}}^{(k)})$.

The major difficulty with this strategy is that it requires one to obtain the ABC posteriors $\tilde{p}_{\varvec{\xi}}({\varvec{\vartheta}}|{\mathbf{z}}^{(k)})$ for $k = 1,\ldots ,K$ at each design point ${\varvec{\xi}}$, which is typically computationally prohibitive.

#### Utility function estimation using ABC rejection sampling

One solution to the problem of having to quickly re-compute the ABC posteriors $\tilde{p}_{\varvec{\xi}}({\varvec{\vartheta}}|{\mathbf{z}}^{(k)})$ for each ${\mathbf{z}}^{(k)} \in Z$ is to simulate a large sample $S_{\varvec{\xi}} = \{{\mathbf{s}}({\mathbf{z}}_r({\varvec{\xi}})),{\varvec{\vartheta}}_r\}_{r=1}^R$ from $p_{\varvec{\xi}}({\mathbf{z}},{\varvec{\vartheta}})$ for a given design ${\varvec{\xi}}$ and to construct the ABC posterior for each ${\mathbf{z}}^{(k)} \in Z$ as a subset of $S_{\varvec{\xi}}$. Those parameter values ${\varvec{\vartheta}}_r$ where the corresponding ${\mathbf{z}}_r$ is in a ${\varepsilon} _k$-neighborhood of ${\mathbf{z}}^{(k)}$, i.e. where $d({\mathbf{z}}^{(k)},{\mathbf{z}}_r) \le {\varepsilon} _k$, constitute the ABC posterior sample. Denoting the corresponding index set by $R_{k} = \{r \in \{1,\ldots ,R\}: d({\mathbf{z}}^{(k)},{\mathbf{z}}_r) \le {\varepsilon} _k\}$, a sample from the ABC posterior $\tilde{p}_{\varvec{\xi}}({\varvec{\vartheta}}|{\mathbf{z}}^{(k)})$ can be obtained by the following rejection sampling algorithm (cf. Algorithm 1):

1. Compute the discrepancies $d({\mathbf{z}}^{(k)},{\mathbf{z}}_r) = d_s({\mathbf{s}}({\mathbf{z}}^{(k)}),{\mathbf{s}}({\mathbf{z}}_r))$ for all particles $r = 1,\ldots ,R$.
2. Accept $${\varvec{\vartheta}}_r$$ if $$r \in R_{k}$$.

Fixing ${\varepsilon} _k$ in advance has the drawback that the ABC sample size $R_{ABC} = {\text {card}}(R_k)$ cannot be controlled. Hence, for practical purposes, it is more convenient to fix $R_{ABC}$, at the expense of having no direct control over the tolerance level ${\varepsilon} _k$, which then results as the $R_{ABC}$ smallest discrepancy $d({\mathbf{z}}^{(k)},{\mathbf{z}}_r)$.

If computer memory permits, it can be useful to pre-simulate the summary statistics ${\mathbf{s}}({\mathbf{z}}_r({\varvec{\xi}}))$ for all possible designs ${\varvec{\xi}}\in \Xi$, so that $S = \{S_{\varvec{\xi}}; \forall \, {\varvec{\xi}}\in \Xi \}$ is available prior to the optimization step. This strategy may help to reduce the overall simulation effort if redundancies between different designs can be exploited. As a further advantage, pre-simulation of the summary statistics for all possible designs permits the application of simulation-based optimal design techniques such as the MCMC sampler of Müller (1999), which is pursued in Drovandi and Pettitt (2013). However, the necessity to store all summary statistics for all designs limits the number of possible candidate designs ${\varvec{\xi}}$ over which to optimize. The number of candidate designs which may be considered depends on the number of distinct summary statistics for each candidate design, the desired ABC accuracy, and the storage capacities.

#### Utility function estimation using importance weight updates

An alternative strategy to obtain a sample from the approximate posterior distribution $\tilde{p}_{\varvec{\xi}}({\varvec{\vartheta}}|{\mathbf{z}}^{(k)})$ is based on importance sampling, see Sect. 3.1. We assume that a weighted sample from the prior distribution, $\{{\varvec{\vartheta}}_r,W_r\}_{r=1}^R$, is available. The goal is to update the weights such that the weighted sample $\{{\varvec{\vartheta}}_r,W_r^{(k)}\}_{r=1}^R$ approximates the ABC posterior distribution $\tilde{p}_{\varvec{\xi}}({\varvec{\vartheta}}|{\mathbf{z}}^{(k)})$. If ${\varvec{\vartheta}}_1,\ldots ,{\varvec{\vartheta}}_R$ is an i.i.d. sample from the prior $p({\varvec{\vartheta}})$, all weights are equal to $W_r = 1/R$. However, the weights might also differ, e.g. when information from previous observations ${\mathbf{z}}_0$ is used to generate an ABC importance sample from the posterior conditioning on ${\mathbf{z}}_0$.

Following Del Moral et al. (2012), we define the ABC target posterior as

3.5 $$\begin{aligned} \tilde{p}_{\varvec{\xi}}({\varvec{\vartheta}},{\mathbf{z}}^{*}_{1:M}|{\mathbf{z}}^{(k)})\propto & {} \left[ \frac{1}{M} \sum _{m=1}^M K_{{\varepsilon} _k}(d({\mathbf{z}}^{(k)},{\mathbf{z}}^{*}_m)) \right] \times \nonumber \\&\left[ \prod _{m=1}^M p_{\varvec{\xi}}({\mathbf{z}}^{*}_m|{\varvec{\vartheta}}) \right] p({\varvec{\vartheta}}), \end{aligned}$$

where $\{{\mathbf{z}}^{*}_m; \; m = 1,\ldots ,M\}$ are auxiliary data, and use the importance density

$$ q_{\varvec{\xi}} ({\varvec{\vartheta}},{\mathbf{z}}^{*}_{1:M}|{\mathbf{z}}^{(k)}) = \left[ \prod _{m=1}^M p_{\varvec{\xi}}({\mathbf{z}}^{*}_m|{\varvec{\vartheta}}) \right] p({\varvec{\vartheta}}). $$

Simulating $\{{\mathbf{z}}^{*}_{r,m}; \; m = 1,\ldots ,M\}$ from $p_{\varvec{\xi}}({\mathbf{z}}^{*}|{\varvec{\vartheta}}_r)$, unnormalized posterior weights of ${\varvec{\vartheta}}_r$ can be estimated by

$$ w_r^{(k)} \propto W_r \sum _{m=1}^M K_{{\varepsilon} _k}(d({\mathbf{z}}^{(k)},{\mathbf{z}}^{*}_{r,m})). $$

It is essential to select $M \gg 1$, as otherwise most of the weights would be close or even equal to zero, leading to a very small effective sample size. As noted in Del Moral et al. (2012), the ABC posterior given in (3.5) has the advantage that

$$ \frac{1}{M} \sum _{m=1}^M K_{{\varepsilon} _k}({\mathbf{z}}^{(k)}|{\mathbf{z}}^{*}_m,{\varvec{\vartheta}}) \rightarrow \int \; K_{{\varepsilon} _k}(d({\mathbf{z}}^{(k)},{\mathbf{z}}^{*})) \; p_{\varvec{\xi}}({\mathbf{z}}^{*}|{\varvec{\vartheta}}) \; {\mathrm{d}} {\mathbf{z}}^{*}$$

for $M \rightarrow \infty$, and hence the sampler is similar to the “marginal” sampler which samples directly from the marginal ABC posterior (3.1).

Just as for the ABC rejection strategy described above, creating the sample $S_ {\varvec{\xi}}^* = \{\{{\mathbf{s}}({\mathbf{z}}^{*}_{r,m}({\varvec{\xi}}))\}_{m=1}^M, {\varvec{\vartheta}}_r \}_{r=1}^R$ in advance can speed up the computations considerably, because $S_ {\varvec{\xi}}^*$ can be re-used to compute $u_{LF}({\mathbf{z}}^{(k)},{\varvec{\xi}})$ for each ${\mathbf{z}}^{(k)}$ sampled from $p_{\varvec{\xi}}({\mathbf{z}})$. It may also be convenient to compute the summary statistics for all design points at once, see the corresponding remarks in Sect. 3.3.1.

Moreover, also similar to Sect. 3.3.1, it is preferable to fix the target ${\text{ESS}}$ instead of selecting the tolerance level ${\varepsilon}$, as the effective sample size may vary substantially between the ABC posterior samples for the different ${\mathbf{z}}^{(k)}$ when the same tolerance level ${\varepsilon}$ is used for all $k = 1,\ldots ,K$. Therefore, we choose a target value for the ${\text{ESS}}$ and adjust ${\varepsilon} _k$ in each step to produce ABC posterior samples with an ${\text{ESS}}$ close to the target value.

For a pre-simulated sample $S^*_ {\varvec{\xi}}$, a fast and flexible sampling scheme targeting a specific effective sample size in each step $k = 1,\ldots ,K$ can be implemented using a uniform kernel, $K_{{\varepsilon} _k}(d({\mathbf{z}}^{(k)},{\mathbf{z}}^{*}_{r,m})) = {\mathbb{I}}(d({\mathbf{z}}^{(k)},{\mathbf{z}}^{*}_{r,m}) \le {\varepsilon} _k)$. Then the weight for particle $r$ is proportional to its prior weight multiplied by the number of simulated data $\{{\mathbf{z}}^{*}_{r,m}\}_{m=1}^M$ with a discrepancy to ${\mathbf{z}}^{(k)}$ below ${\varepsilon} _k$, i.e.

3.6 $$ w_r^{(k)} = W_r \sum _{m=1}^M {\mathbb{I}}(d({\mathbf{z}}^{(k)},{\mathbf{z}}^{*}_{r,m}) \le {\varepsilon} _k). $$

To roughly keep a defined ESS for each $k$ we proceed as follows. Let $D_{r,k} = \{d({\mathbf{z}}^{(k)},{\mathbf{z}}^{*}_{r,m})\}_{m=1}^M$ denote the set of discrepancies between ${\mathbf{z}}^{(k)}$ and ${\mathbf{z}}^{*}_{r,m}$ and let $D_k= \{D_{r,k}\}_{r=1}^R$. For each $k$, the set $D_k$ can be searched for the tolerance level ${\varepsilon} _k$ which yields the best approximation to the target ${\text{ESS}}$. The weights are computed from (3.6) and the ESS results from (3.4). The advantage of using a uniform kernel is that the weight $w_r^{(k)}$ only depends on the number of elements in $D_{r,k}$ which are not larger than ${\varepsilon} _k$. Binary search algorithms can be applied on the sorted set $D_{r,k}$ to determine this number in an efficient manner.

## Spatial extremes

In this section we review some basic concepts of extreme value theory which are needed in our application in Sect. 5.

### Max-stable processes

The joint distribution of extreme values at given locations $x_1,\ldots , x_D \in X$ can be modeled as marginal distribution of max-stable processes on $X \subset {\mathbb {R}}^p$. Max-stable processes arise as the limiting distribution of the maxima of i.i.d. random variables on $X$, see de Haan (2004) for a concise definition. A property of max-stable processes which allows convenient modeling is that their multivariate marginals are members of the class of multivariate extreme value distributions, and univariate marginals have a univariate generalized extreme value (GEV) distribution.

The cumulative distribution function of the univariate GEV distribution is given as

$$ G(z) = \exp \left[ -\left( 1 + \zeta \frac{z - \mu}{\sigma} \right) ^{-1/\zeta}_+ \right] , $$

where $\mu , \, \sigma > 0$, and $\zeta$ are the location, scale, and shape parameters, respectively, and $z_+ = \max (z,0)$. The GEV distribution with parameters $\mu =\sigma =\zeta =1$ is called the unit Fréchet distribution. Any GEV random variable $Z$ can be transformed to unit Fréchet by the transformation

4.1 $$ t(Z)= \left( 1 + \zeta \frac{Z - \mu}{\sigma} \right) ^{1/\zeta}. $$

This property allows to focus on max-stable processes with unit Fréchet margins when the dependence structure is of interest. Hence we assume that all univariate marginal distributions are unit Fréchet in what follows.

### Dependence structure of max-stable processes 

The multivariate distribution of a max-stable process with unit Fréchet margins at the locations $x_1,\ldots ,x_k$ has the form

4.2 $$ {\mathrm{P}}(Z(x_1) \le z_1,\ldots ,Z(x_k) \le z_k) = \exp \left( -V(z_1,\ldots ,z_k) \right) . $$

The function $V$ is a homogeneous function of order $-1$,

4.3 $$ V(t z_1,\ldots , t z_k)=t^{-1}V(z_1,\ldots , z_k), $$

and is called the exponent measure (Pickands 1981). The dependence structure of a stationary max-stable process can be modeled via one of its spectral representations. These representations are useful as they often allow for an interpretation of the max-stable process in terms of maxima of underlying processes (see e.g. Smith (1990), Schlather (2002), or Davison et al. (2012)) and make it possible to devise sampling schemes for many max-stable processes.

Here we will consider the model introduced by Schlather (2002). Let $\{S_i\}_{i \in {\mathbb{N}}}$ be a Poisson process on $(0,\infty )$ with intensity ${\mathrm{d}}s/s^2$ and $\{Y_i(x)\}_{i \in {\mathbb{N}}}$ be independent replicates of a stationary process $Y(x)$ on ${\mathbb{R}}^p$ with ${\mathrm{E}}\big (\max (0,Y_i(x))\big ) = 1$. Then

$$ Z(x) = \max _{i} S_i \max (0,Y_i(x)) $$

is a stationary max-stable process with unit Fréchet margins. In the Schlather model, $Y(x)$ is specified as a Gaussian process. If the Gaussian random field is isotropic, it has the correlation function $\rho (h; {\varvec{\phi}})$, where $h = \Vert x_1 - x_2\Vert$ is the distance between two points $x_1$ and $x_2$ and $\varvec{\phi}$ denotes the parameters of $\rho$. The correlation function has to be chosen from one of the correlation families for Gaussian processes, e.g. *Whittle–Matérn*, *Cauchy*, or *powered exponential*. For the Schlather model, a closed form of the likelihood exists only for $k = 2$ points.

#### Extremal coefficients

A useful summary measure for extremal dependence is given by the extremal coefficients, which are defined via the marginal cdfs of a max-stable process. From (4.2) and (4.3), the joint cdf of $Z_1(x_1),\ldots ,Z_k(x_k)$ at $z_1=\cdots =z_k=z$ is given as

$$\begin{aligned} {\mathrm{P}}(Z(x_1) \le z,\ldots ,Z(x_k) \le z)&=\exp \left( -\frac{V(1,\ldots ,1)}{z} \right) =\\&= \exp \left( -\frac{\theta (x_1,\ldots ,x_k)}{z} \right) . \end{aligned}$$

$\theta (x_1,\ldots ,x_k)$ is called the $k$-point extremal coefficient between the locations $x_1,\ldots ,x_k$. Though the extremal coefficients between all the sets of $k$ points ($k=2,\ldots ,D$) contain a substantial amount of the information on the dependence structure of the max-stable process, they are not sufficient to characterize the whole process.

Given $n$ block maxima $z_1(x_i),\ldots ,z_n(x_i)$ observed at each of the points $x_i \in \{x_1,\ldots ,x_k\}$, Erhardt and Smith (2012) propose to estimate the $k$-point extremal coefficient by the simple estimator

4.4 $$ {\hat{\theta}}(x_{1},x_{2},\ldots , x_{k}|{\mathbf{z}}) = \frac{n}{\sum _{i=1}^n 1/\max (z_i(x_{1}),z_i(x_{2}), \ldots , z_i(x_{k}))}, $$

where $${\mathbf{z}}= \{z_i(x_j); \, j = 1,\ldots ,k; \, i = 1,\ldots ,n\}$$.

## Application

We illustrate our likelihood-free methodology on an application where the aim is to find the optimal design for estimating the parameters characterizing the dependence of spatial extreme values. As our example is meant to illustrate the basic methodology, we use a simple design setting.

**Fig. 1:**
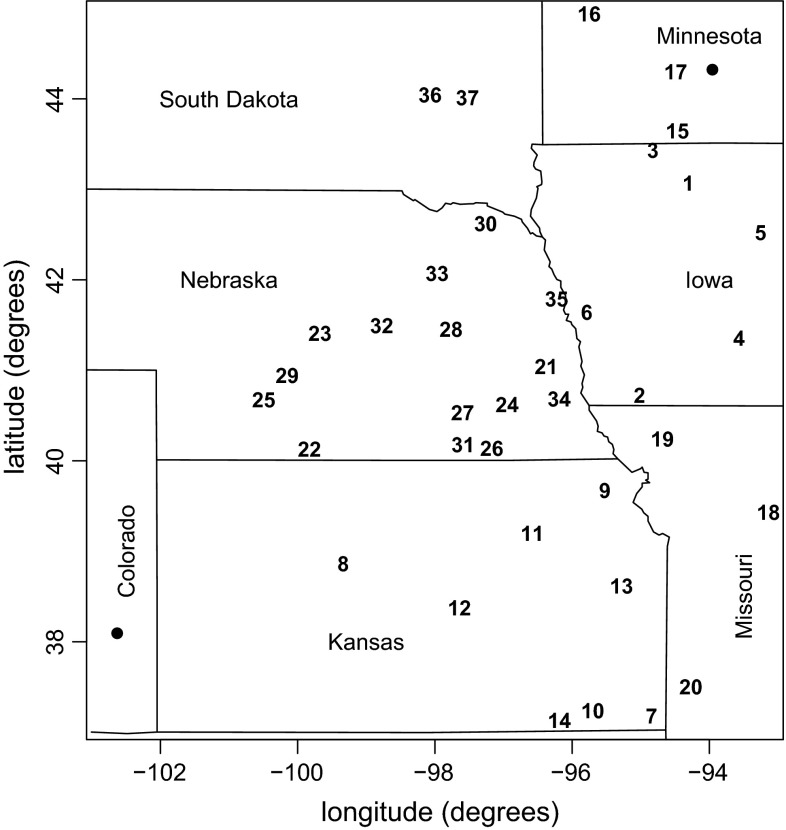
Locations and numbers of weather stations in the Midwest region of the USA

The problem we consider is inspired by the paper of Erhardt and Smith (2014), who use data on maximum annual summer temperatures from 39 sites in the Midwest region of the USA for pricing weather derivatives. Figure 1 shows a map of the 39 weather stations. The dots (bottom left and top right) indicate the two stations with the largest mutual distance, which we will include in each design. Our goal is to determine which of the remaining 37 stations, indicated by the numbers 1–37, should be kept to allow optimal inference for the spatial dependence parameters. Thus we intend to find the optimum three-point design.

We specify the spatial extremes model as a Schlather model (Schlather 2002) with the Whittle–Matérn correlation function. The Schlather model requires us to select a correlation function, which is also part of the model choice. However, the Whittle–Matérn correlation function is a quite flexible correlation function. It is specified as

$$\begin{aligned} \rho (h; c, \lambda , \kappa ) = {\left\{ \begin{array}{ll} 1, &{} h = 0\\ c \; \frac{2^{1-\kappa}}{\Gamma (\kappa )} \; \left( \frac{h}{\lambda} \right) ^{\kappa} \; K_{\kappa}\left( \frac{h}{\lambda} \right) , &{} h > 0 \end{array}\right.} \end{aligned}$$

where $K_{\kappa}$ is the modified Bessel function of the second kind of order $\kappa$, and $0 \le c \le 1$, $\lambda > 0$, $\kappa > 0$. We fix the partial sill parameter $c$ at $c = 1$ (which is a standard choice, see the applications of max-stable processes in Davison et al. 2012) and the smooth parameter $\kappa$ at $\kappa = 0.5$. The smooth parameter $\kappa$ is fixed, since widely different values for $\lambda$ and $\kappa$ can result in similar values for the correlation function, making joint inference for both parameters more difficult.

As utility function we choose the posterior precision of the range parameter $\lambda$, which is the only parameter to be estimated, i.e.

$$ u({\mathbf{z}},{\varvec{\xi}}) = 1/\det \left( {\text {Var}}_{\varvec{\xi}} ({\varvec{\vartheta}}|{\mathbf{z}}) \right) = 1/{\text {Var}}_{\varvec{\xi}} (\lambda |{\mathbf{z}}). $$

Following Erhardt and Smith (2012), we use the tripletwise extremal coefficient for each three-point design as summary statistic for ABC inference.

For a three-point design, the gain in information from the prior to the posterior distribution will be very low unless many observations are available. Therefore, we obtain the optimal design for samples of size $n=1000$, so that we are able to clearly identify differences between the expected posterior precision values for different designs. For practical purposes, the three-point designs can be sequentially augmented by further design points. One can stop when the amount of data available in practice is sufficient to exceed a desired minimum expected posterior precision.

In Sect. 5.1, we compare ABC rejection and ABC importance sampling for likelihood-free optimal design for the case where a standard uniform prior distribution is specified for $\lambda$. In Sect. 5.2, we go one step further and additionally incorporate information from prior observations. In our case, data from 115 years collected at the 39 stations were used to estimate an ABC posterior distribution for the range parameter. This posterior distribution was then used as parameter distribution in an importance weight update algorithm to determine the optimal three-point design for future inference.

### Comparison of likelihood-free design algorithms

#### Settings

In the case where we have no prior observations, we assumed a uniform $U[2.5,17.5]$ prior for the parameter $\lambda$, which is similar as in Erhardt and Smith (2012). This prior is meant to cover all plausible range parameter values, since the largest inter-site distance is $10.68$, the smallest is $0.36$. Its density is displayed as dashed line in Fig. 3.

The goal is to find the design $${\varvec{\xi}}$$ for which $$\hat{U}({\varvec{\xi}}) = $$$$K^{-1} \sum _{k=1}^K \hat{u}_{LF}({\mathbf{z}}^{(k)},{\varvec{\xi}})$$ is maximal (see Eq. (2.2)), where we set $$K = 2000$$, $$\hat{u}_{LF}({\mathbf{z}}^{(k)},{\varvec{\xi}}) = 1/\widehat{\text {Var}}_{\varvec{\xi}} (\lambda |{\mathbf{z}}^{(k)})$$, and $${\mathbf{z}}^{(k)} \sim p_{\varvec{\xi}}({\mathbf{z}})$$ are samples of size $$n=1000$$ from the prior predictive distribution. We now give details for both the rejection sampling algorithm and the importance weight update algorithm.

For the ABC rejection sampling algorithm (see Sect.  3.3.1), as a first step we pre-simulated samples

$$ S_{\varvec{\xi}} = \{{\mathbf{s}}({\mathbf{z}}_r({\varvec{\xi}})),{\varvec{\vartheta}}_r \}_{r=1}^R = \{\hat{\theta}({\mathbf{x}}_{\varvec{\xi}}|{\mathbf{z}}_r),\lambda _r \}_{r=1}^R $$

of size $R = 5 \cdot 10^6$ for all ${\text {card}}(\Xi )= 37$ designs by sampling $\lambda _r$ from the prior and ${\mathbf{z}}_r|\lambda _r$ (having size $n=1000$) from the Schlather model. As a summary statistic, ${\mathbf{s}}(.)$, we use the estimated tripletwise extremal coefficient ${\hat{\theta}}({\mathbf{x}}_{\varvec{\xi}}| {\mathbf{z}}_r)$ computed according to Formula (4.4) for the simulated observations ${\mathbf{z}}_r$ at the design coordinates ${\mathbf{x}}_{\varvec{\xi}}$.

As the next step, for each design ${\varvec{\xi}}\in \Xi$, we simulated observations ${\mathbf{z}}^{(k)}$ ($k=1,\ldots , K=2000$) and computed the tripletwise extremal coefficient ${\hat{\theta}}({\mathbf{x}}_{\varvec{\xi}}| {\mathbf{z}}^{(k)})$. The ABC posterior sample was formed by those 500 (0.01 %) elements of $S_{\varvec{\xi}}$ with the lowest absolute difference $|\hat{\theta}({\mathbf{x}}_{\varvec{\xi}}| {\mathbf{z}}^{(k)})-\hat{\theta}({\mathbf{x}}_{\varvec{\xi}}| {\mathbf{z}}_r)|$. This ABC posterior sample was then used to compute $\hat{u}_{LF}({\mathbf{z}}^{(k)},{\varvec{\xi}}) = 1/\widehat{\text {Var}}_{\varvec{\xi}} (\lambda |{\mathbf{z}}^{(k)})$ for each $k = 1,\ldots ,K$.

For the importance weight update algorithm, we generated the pre-simulated sample $S_{\varvec{\xi}}^*$ as follows: a sample $\{\lambda _r\}_{r=1}^R$ of size $R = 2000$ was obtained from the prior distribution. For each $\lambda _r$, a collection of $M=4000$ samples $\{{\mathbf{z}}^{*}_{r,m}; \; m = 1,\ldots ,M\}$ from the Schlather model was generated and the tripletwise extremal coefficients were computed for all designs. Each ${\mathbf{z}}^{*}_{r,m}$ consisted of $n=1000$ observations.

In the Monte Carlo integration step, for each design ${\varvec{\xi}}$, the samples ${\mathbf{z}}^{(k)}$ ($k=1,\ldots ,K=2000$) of size $n = 1000$ were generated and the normalized importance weights $W_r^{(k)} = w_r^{(k)} / \left( \sum _{r=1}^R w_r^{(k)} \right)$ were computed from (3.6), where the absolute difference between the corresponding tripletwise extremal coefficients was used as discrepancy $d({\mathbf{z}}^{(k)},{\mathbf{z}}^{*}_{r,m})$. The weighted ABC posterior sample $\{\lambda _r,W_r^{(k)}\}_{r=1}^R$ was used to estimate $\hat{u}_{LF}({\mathbf{z}}^{(k)},{\varvec{\xi}}) = 1/\widehat{\text {Var}}_{\varvec{\xi}} (\lambda |{\mathbf{z}}^{(k)})$. For each $k$, we aimed to obtain samples from the ABC posterior with target ESS = 100.

#### Results

All computations were performed on the SGI Altix 4700 SMP system using 20 nodes in parallel. For the ABC rejection method, it took about 28 h to generate the pre-simulated sample of length $R = 5 \cdot 10^6$, which required roughly 1.35 GB. The Monte Carlo integration procedure, where the utility functions for the $K = 2000$ samples from the prior predictive distribution are evaluated and the average is computed, needed about 2.6 h. For the importance weight update method, the pre-simulated sample of length $R \cdot M = 2000 \cdot 4000 = 8 \cdot 10^6$ was generated in 46 h and produced a file of size 2.06 GB. The Monte Carlo integration took about 5.5 h.

**Fig. 2:**
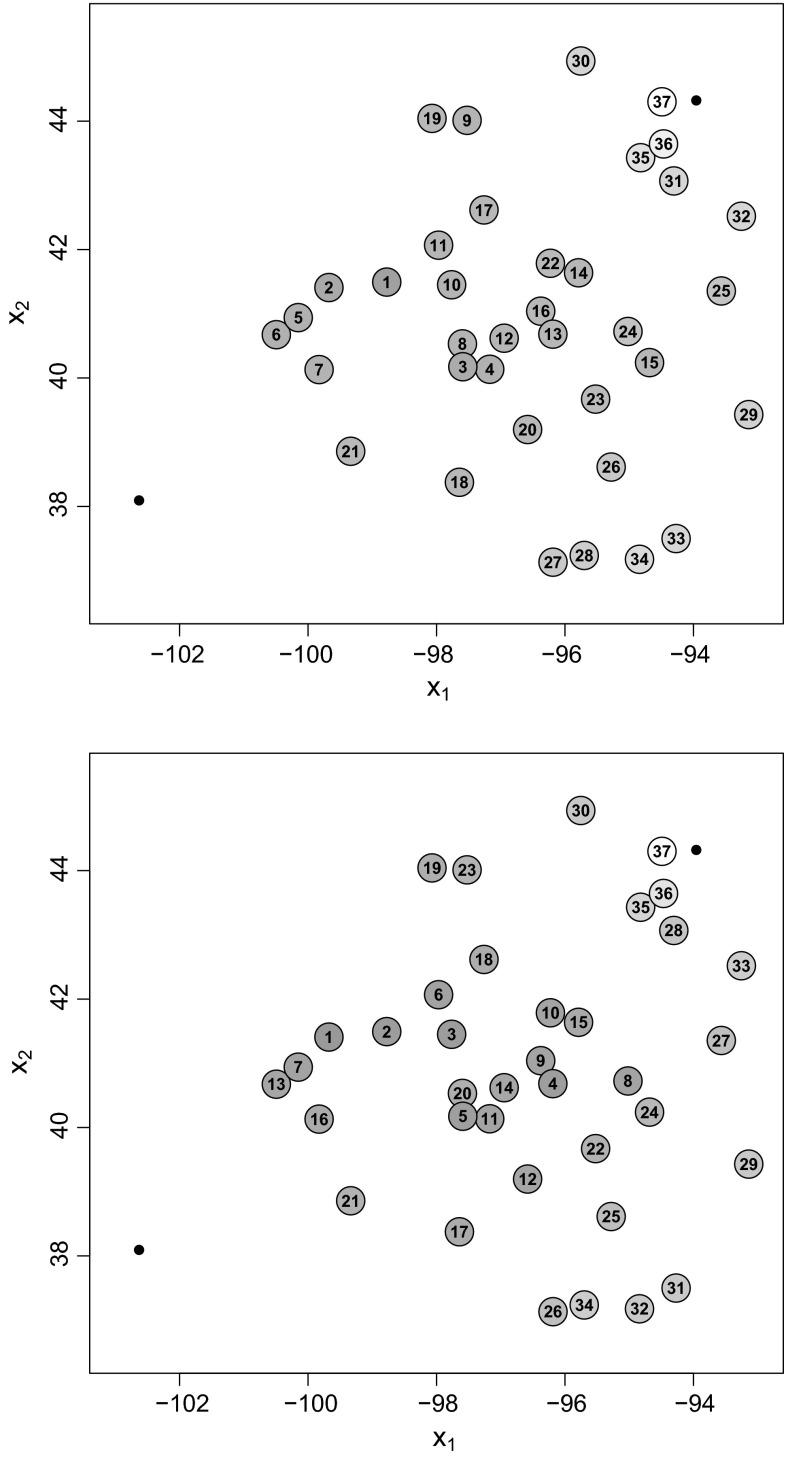
Rankings of expected utility criterion $\hat{U}({\varvec{\xi}}) = K^{-1} \sum _{k=1}^K \hat{u}_{LF}({\mathbf{z}}^{(k)},{\varvec{\xi}})$, where $\hat{u}_{LF}({\mathbf{z}}^{(k)},{\varvec{\xi}}) = 1/\widehat{\text {Var}}_{\varvec{\xi}} (\lambda |{\mathbf{z}}^{(k)})$ is the ABC posterior precision utility of the range parameter, when the uniform prior $\lambda \sim U[2.5,17.5]$ is used: *top* ABC rejection method, *bottom* importance weight update method

Figure 2 shows the results for both methods for one particular simulation run. Designs are indicated by circles, where the number denotes the rank of the design with respect to the expected utility criterion, and the two fixed stations are indicated by black dots. The ranking of the designs is additionally visualized by the filling intensity: the circle for the design with the highest criterion value across both methods is darkest ($\hat{U}({\varvec{\xi}}_{\max}) = 0.604$ for station 23 using the importance weight update method), whereas the design with the lowest criterion value across both methods is white ($\hat{U}({\varvec{\xi}}_{\min}) = 0.362$ for station 17 using the ABC rejection method). The gray levels of all the other circles are in between these two extreme levels in proportion to their criterion values.

The results of both methods correspond closely. There are only negligible differences with respect to the estimated design criterion values for the large majority of design points which lie in the middle between the two fixed stations, indicated by similar filling intensities in Fig. 2. On the other hand, rankings can differ considerably due to Monte Carlo error. However, we observe that differences in rankings occur for designs with approximately the same expected utility values. Therefore, all these designs are almost equally well-suited for conducting experiments, so differences in rankings are of minor interest. However, the expected utilities for the design points close to the fixed design point in the upper right corner as well as the design points in the lower right, which are far away from either fixed station, have notably lower expected utility values.

We varied the target effective sample sizes for both the ABC rejection method and the importance weight update method. The ABC rejection method was also run using increased ABC sample sizes of $50000$ and $500000$. We could not observe any discernible effects on the general pattern of criterion orderings. The same can be said about the importance weight update method, where we computed the rankings for different target effective sample sizes between $100$ and $500$. The details are provided in Section 1 of Online Resource 1.

### Incorporating information from prior observations

As briefly mentioned in Sect. 3.3.2, information from prior observations can easily be incorporated to estimate the design criteria using the importance weight update algorithm. Information from prior observations can be processed by any suitable ABC algorithm to obtain an ABC posterior sample for the parameters, which serves as “input prior” sample in the importance weight update algorithm.

We illustrate the incorporation of information from prior data by using the data previously analyzed in Erhardt and Smith (2014). The data set contains maximum summer (June 1–August 31) temperature records collected at the 39 stations from 1895 to 2009 (115 observations). The daily data can be downloaded from the National Climatic Data Center (http://cdiac.ornl.gov/ftp/ushcn_daily). The block maximum for year $t$ at location $x$ is obtained by computing $z_t(x) = \max (y_{t,1}(x),\ldots ,y_{t,92}(x))$, where $\{y_{t,i}(x)\}_{i=1}^{92}$ denotes the 92 maximum daily temperature observations in summer. Erhardt and Smith (2014) performed checks of the GEV and Schlather model assumptions for this data set and concluded that the Schlather model is appropriate.

Following Erhardt and Smith (2014), we transformed the original data to unit Fréchet scale at each location using Eq.  (4.1), where estimates of the marginal GEV parameters $\mu (x)$, $\sigma (x)$, and $\zeta (x)$ at location $x$ were plugged in.

We specified a uniform $U[0,20]$ prior for $\lambda$ and applied ABC rejection sampling, see Algorithm 1, to derive the ABC posterior for $\lambda$. As in Erhardt and Smith (2012), we used a discrepancy function based on tripletwise extremal coefficients. We note here that with data from 39 stations, there are $9139$ tripletwise extremal coefficients, which requires a more sophisticated discrepancy function compared to that in Sect.  5.1. Dimension reduction was achieved by clustering the extremal coefficients according to the inter-site distances into 100 clusters. Only the average values within each cluster were used as summary statistics. Finally, the discrepancy between two vectors of summary statistics was computed by the Manhattan distance, for details see Erhardt and Smith (2012).

**Fig. 3:**
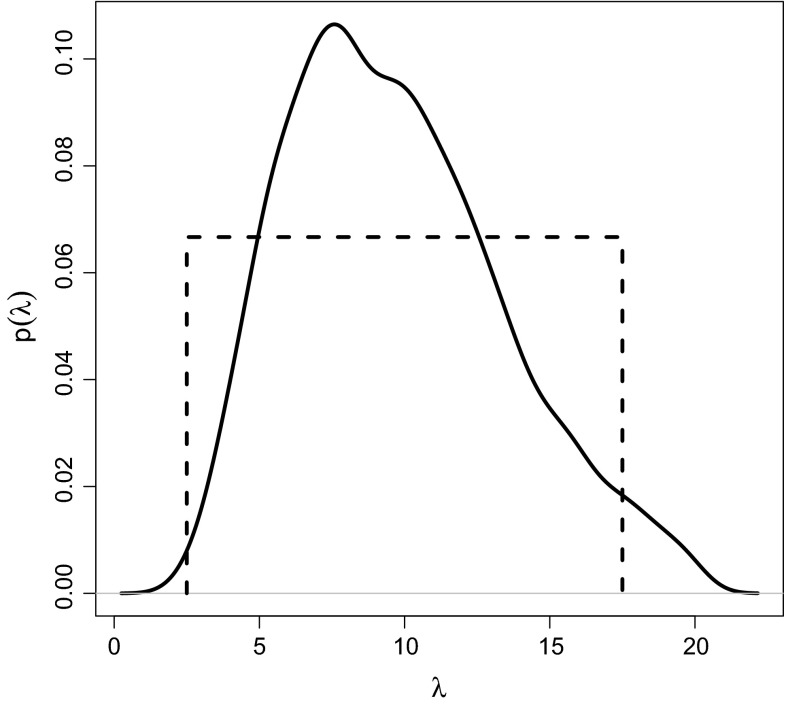
*Solid line* Kernel density estimate of ABC posterior obtained by combining information from previous observations with prior $\lambda \sim U[0,20]$, used as “input prior” in Sect. 5.2. *Dashed line* $U[2.5,17.5]$ prior used in Sect. 5.1

We generated a sample $\{{\mathbf{z}}_q,\lambda _q\}_{q=1}^Q$ of size $Q=10^7$ from $p_{\varvec{\xi}}({\mathbf{z}}|\lambda ) p(\lambda )$ for the design including all 39 points and kept only those $R=2000$ (0.02 %) draws yielding the smallest values of the discrepancy to the original sample. The resulting posterior distribution is shown in Fig. 3 (solid line). This distribution is more informative about the parameter than the flat uniform prior used in Sect. 5.1 (dashed line).

The ABC posterior sample was then used as prior sample in the importance weight update algorithm from Sect. 3.3.2, with the same settings as in Sect. 5.1.1: for each $\lambda _r$ ($r=1,\ldots ,R$), we simulated $M = 4000$ samples of size $n=1000$ taken at the 39 sites and stored the tripletwise extremal coefficients as summary statistics. To compute $\hat{U}({\varvec{\xi}})$ for each ${\varvec{\xi}}\in \Xi$, we generated $K=2000$ samples ${\mathbf{z}}^{(k)}$ (also of size $n=1000$) from the prior predictive distribution. The simulation times were very similar to those of the importance weight update method for the uniform prior in Sect. 5.1.

**Fig. 4:**
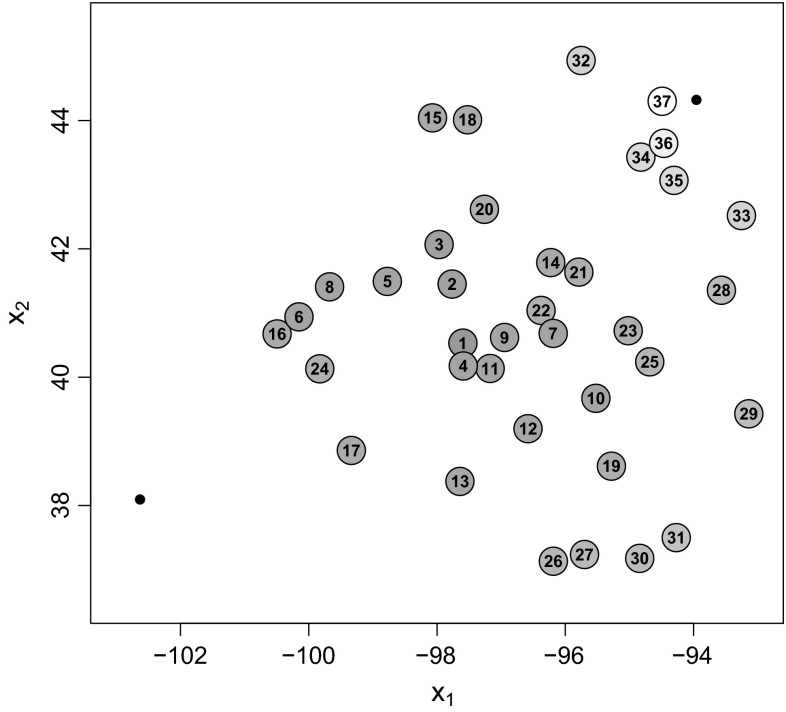
Rankings of expected utility criterion $\hat{U}({\varvec{\xi}}) = K^{-1} \sum _{k=1}^K \hat{u}_{LF}({\mathbf{z}}^{(k)},{\varvec{\xi}})$, where $\hat{u}_{LF}({\mathbf{z}}^{(k)},{\varvec{\xi}}) = 1/\widehat{\text {Var}}_{\varvec{\xi}} (\lambda |{\mathbf{z}}^{(k)})$ is the ABC posterior precision utility of the range parameter, when using the importance weight update method with the ABC posterior displayed in Fig. 3 (*solid line*) as input prior

Figure 4 shows the ranking of the design points when the ABC posterior for $\lambda$ is used as prior for the importance weight update algorithm. The gray levels correspond to the criterion values of the design points relative to the maximum value $\hat{U}({\varvec{\xi}}_{\max}) = 0.471$ (rank 1 at station 27, dark grey) and the minimum value $\hat{U}({\varvec{\xi}}_{\min}) = 0.31$ (rank 37 at station 17, white). The ranking exhibits the same general pattern as those in Fig. 2 for the $U[2.5,17.5]$ uniform prior. Points very close to one of the fixed points and points very far away from either fixed point have a lower expected utility than the design points in the middle.

**Fig. 5:**
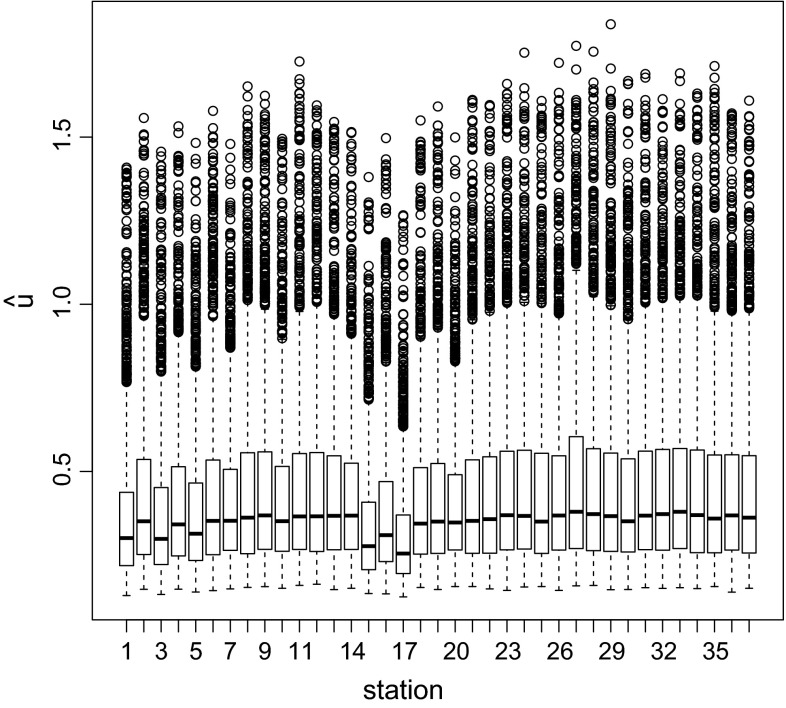
Boxplots of the $K = 2000$ ABC posterior precision utility values for the range parameter ($\{\hat{u}_{LF}({\mathbf{z}}^{(k)},{\varvec{\xi}}) = 1/\widehat{\text {Var}}_{\varvec{\xi}} (\lambda |{\mathbf{z}}^{(k)});\; k = 1,\ldots ,K\}$) for all designs when using the importance weight update method with the ABC posterior displayed in Fig. 3 (*solid line*) as input prior. The design (station) numbers correspond to the numbers in Fig. 1

The distribution of the $K = 2000$ simulated utility values $\hat{u}_{LF}({\mathbf{z}}^{(k)},{\varvec{\xi}}) = 1/\widehat{\text {Var}}_{\varvec{\xi}}(\lambda |{\mathbf{z}}^{(k)})$ is displayed in Fig. 5, where the 37 designs are numbered as in Fig. 1. One can see, for example, that stations 15 and 17 in Minnesota, which are situated close to the top right station, have comparably low utility values.

In Section 2 of Online Resource 1, we investigate the effect of the Monte Carlo error on the design rankings in this example by performing several simulation runs. The rankings differ in particular for the designs in the middle. For these, however, the criterion values are very similar.

When we use another pre-simulated sample, only minor shifts in the resulting rankings occur, which indicates that our choice of $R=2000$ and $M=4000$ is sufficient. On the other hand, we observe larger differences between the results if we use different random samples $\{{\mathbf{z}}^{(k)};\; k = 1,\ldots ,K=2000\}$ from the prior predictive distribution. Hence, in our example it would be worthwhile to increase $K$ in order to improve the accuracy of the criterion estimates.

## Conclusion

In this paper we presented an approach for Bayesian design of experiments when the likelihood of the statistical model is intractable and hence classical design, where the utility function is a functional of the likelihood, is not feasible. In such a situation ABC methods can be employed to approximate a Bayesian utility function, which is a functional of the posterior distribution. For a finite design space, the conceptually straightforward approach is to run ABC for each design and each data set ${\mathbf{z}}^{(k)}$, $k = 1,\ldots ,K$, but this will typically be computationally prohibitive.

As we demonstrate here, a useful strategy is to pre-simulate data for a sample of parameter values at each design. Employing ABC rejection sampling or ABC importance sampling then allows to obtain approximations of the utility function. In our application, the importance weight update method turns out to be particularly useful to incorporate information from prior observations. Both methods are also applicable to situations where the likelihood is in principle tractable, but the posterior is difficult or time-consuming to obtain.

A notorious problem of any ABC method is the choice of the summary statistics, as in problems where one will resort to ABC methods typically no sufficient statistics are available, and the quality of the ABC posterior as an approximation to the true posterior critically depends on the summary statistics. The usefulness of the tripletwise extremal coefficient was validated by Erhardt and Smith (2012). It therefore seems appropriate as ABC summary statistic in our application, where the goal is to find the optimal design consisting of three weather stations. For higher-dimensional designs different summary statistics with lower dimension might be more advantageous.

A further drawback of the presented approach is that memory space and/or computing time restrictions will only permit optimization over a rather small number of designs. For a large design space, a stochastic search algorithm, e.g. as in Müller et al. (2004), should be employed.

## Electronic supplementary material

Below is the link to the electronic supplementary material.

(PDF 105 kb)
