# Supplementary material for: Likelihood-free simulation-based optimal design with an application to spatial extremes
Source: Stoch Environ Res Risk Assess. 2015 Apr 12;30:481–92. doi: 10.1007/s00477-015-1067-8 (PMC4981187; doi:10.1007/s00477-015-1067-8)
Supplement: Supplementary file 1 — (PDF 105 kb) [file 477_2015_1067_MOESM1_ESM.pdf]

Online resource 1 for "Likelihood-free simulation-based optimal design with an application to spatial extremes"

published in *Stochastic Environmental Research and Risk Assessment*

by Markus Hainy, Werner G. Müller, and Helga Wagner
Department of Applied Statistics
Johannes Kepler University Linz
Altenberger Strasse 69, 4040 Linz, Austria
e-mail: markus.hainy@jku.at

1 Effect of increasing the effective sample size

We investigate the effect of increasing the target effective sample size (ESS) on the resulting rankings for the ABC rejection method (Figure 1) and the importance weight update method (Figure 2) for the example in Section 5.1 of the main article, where we used the uniform $U[2.5, 17.5]$ prior.

For the ABC rejection method, selecting a target ESS corresponds to setting the number R_{ABC} of accepted particles (see Section 3.3.1 of the main article) to a fixed value. In Figure 1, we compare the rankings for a total of $R = 5 \cdot 10^6$ particles when $R_{ABC} = 500$ (as in section 5.1. of the main article), $R_{ABC} = 5 \cdot 10^4$ and $R_{ABC} = 5 \cdot 10^5$.

For the importance weight update method, Section 3.3.2 in the main article describes how the tolerance levels can be adjusted to achieve weighted posterior samples with an ESS that comes as close as possible to the chosen target ESS. Since there are $R = 2000$ particles from the prior distribution of the parameter, the ESS can assume values between 1 (weight of 1 to one particle) and 2000 (equal weights to all particles). In Section 5.1 of the main article, we choose a target ESS of 100. We now investigate the effect of increasing the target ESS to ESS = 300 and to ESS = 500.

As we used the same seeds for generating the pre-simulated data and the data from the prior predictive distributions for all effective sample sizes, differences in the rankings are solely attributable to the effects of choosing different effective sample sizes.

From Figures 1 and 2 we conclude that the rankings are moderately sensitive to increases of the effective sample size, but the main conclusions drawn in the article still remain valid.

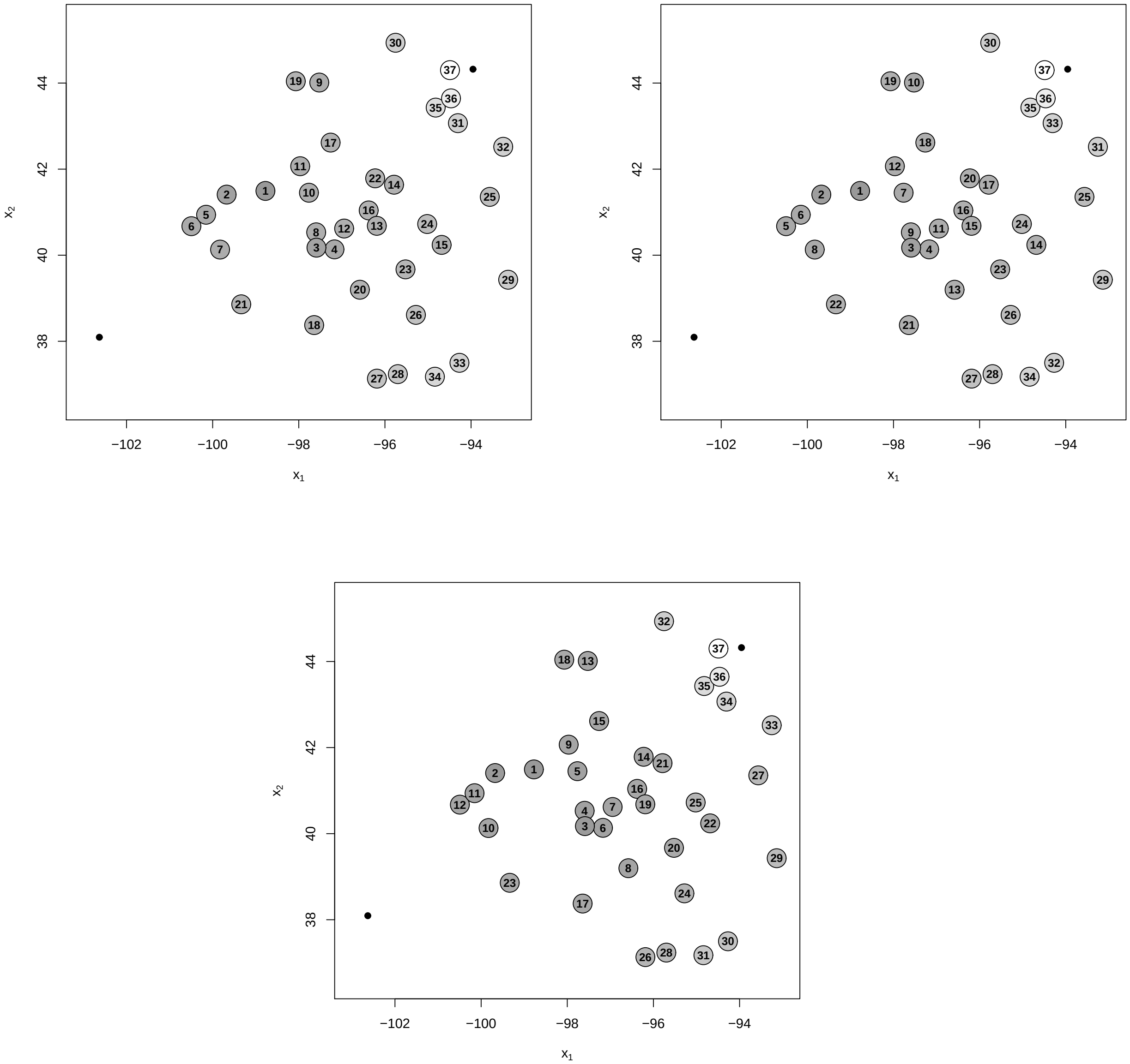

Figure 1: Rankings of expected utility criterion $\hat{U}(\boldsymbol{\xi}) = K^{-1}\sum_{k=1}^{K}\hat{u}_{LF}(\mathbf{z}^{(k)},\boldsymbol{\xi})$, where $\hat{u}_{LF}(\mathbf{z}^{(k)},\boldsymbol{\xi}) = 1/\widehat{\mathrm{Var}}_{\boldsymbol{\xi}}(\lambda|\mathbf{z}^{(k)})$ is the ABC posterior precision utility of the range parameter, when the ABC rejection method with a uniform $U[2.5, 17.5]$ prior is used:
top left: $ESS = 500$, **top right**: $ESS = 5\cdot 10^4$, **bottom**: $ESS = 5\cdot 10^5$

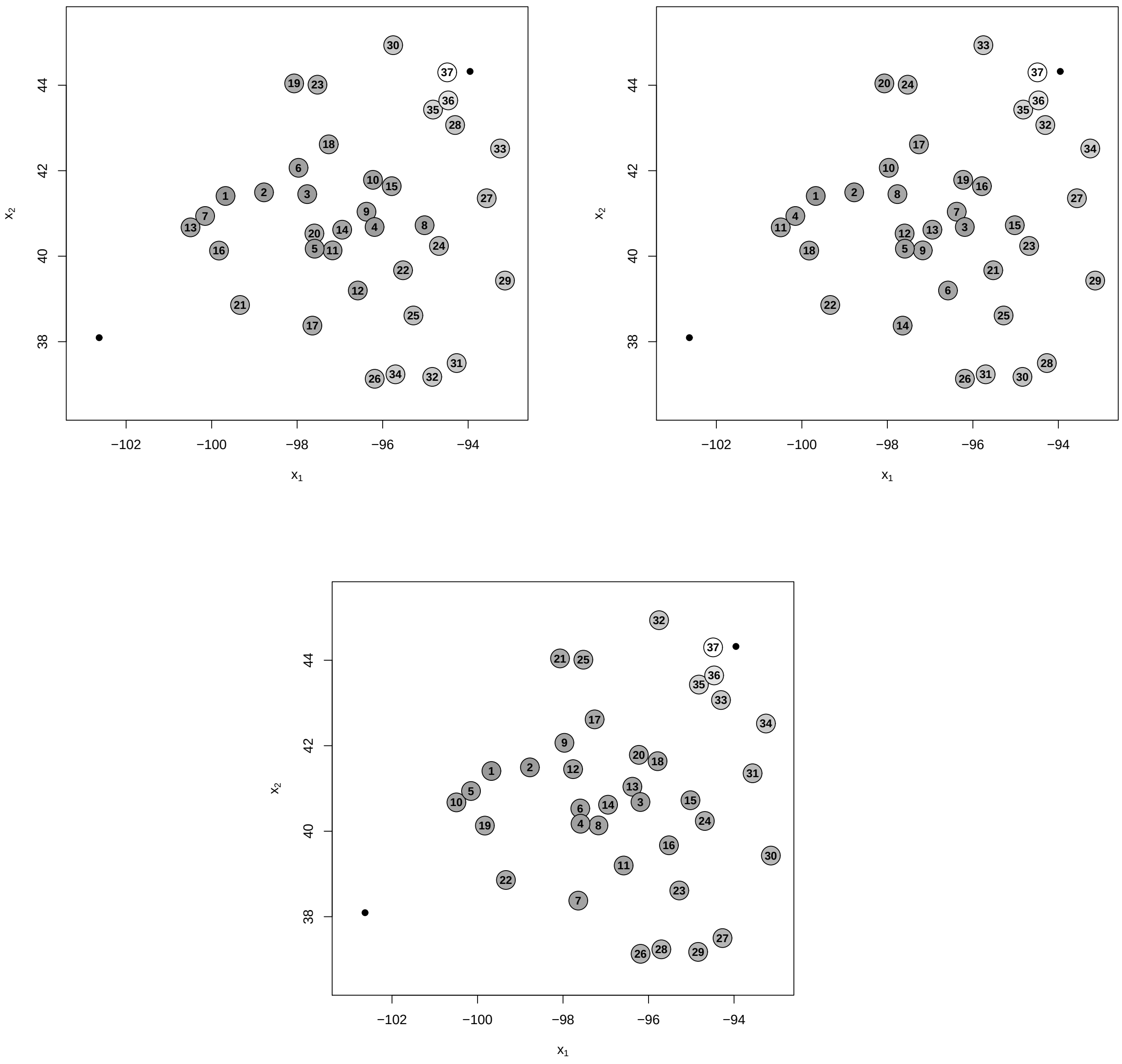

Figure 2: Rankings of expected utility criterion $\hat{U}(\boldsymbol{\xi}) = K^{-1}\sum_{k=1}^{K} \hat{u}_{LF}(\mathbf{z}^{(k)}, \boldsymbol{\xi})$, where $\hat{u}_{LF}(\mathbf{z}^{(k)}, \boldsymbol{\xi}) = 1/\widehat{\mathrm{Var}}_{\boldsymbol{\xi}}(\lambda|\mathbf{z}^{(k)})$ is the ABC posterior precision utility of the range parameter, when the importance weight update method with a uniform $U[2.5, 17.5]$ prior is used:
top left: $ESS = 100$, **top right**: $ESS = 300$, **bottom**: $ESS = 500$

2 Effect of Monte Carlo error

In this section we examine the effect of the Monte Carlo error on the resulting rankings for the example in Section 5.2 of the main article. There we use the importance weight update method with input prior given by the ABC posterior distribution estimated from previous observations. We made a total of eight simulation runs, which are shown in Figures 3 and 4 (Figure 4 being just a continuation of Figure 3).

The columns correspond to two pre-simulated samples S_1^* and S_2^* that were generated using different random seeds. The first column uses the same pre-simulated sample that was also used in the main article.

The rows show the different results when varying the seeds for drawing the sample $\{\mathbf{z}^{(k)};\ k = 1, \dots K = 2000\}$ from the prior predictive distribution during Monte Carlo integration. The random seed used for the first row corresponds to the random seed that was also used in the example in the main article, so that the upper left image in Figure 3 reproduces Figure 4 of the main article.

The maximum criterion value across all designs and simulation runs is 0.472, for which the maximum gray level is attained. Conversely, 0.309 is the smallest criterion value, which corresponds to the minimum gray level (=white). The gray levels of all circles lie between these two extreme values in proportion to their respective criterion values.

From Figures 3 and 4 we can conclude that differences are much smaller across columns (different pre-simulated data sets) than across rows (different data from the prior predictive distribution). This indicates that our pre-simulated sample of size $R \cdot M = 2000 \cdot 4000 = 8 \cdot 10^6$ seems to be sufficiently large to avoid pronounced effects of the Monte Carlo error originating from the randomness of the pre-simulated sample. Therefore, to reduce the Monte Carlo error of the criterion values and rankings in our case, it would be more worthwhile to increase the size K of the prior predictive sample $\{\mathbf{z}^{(k)}\}_{k=1}^{K}$ than to increase the size of the pre-simulated sample S^*.

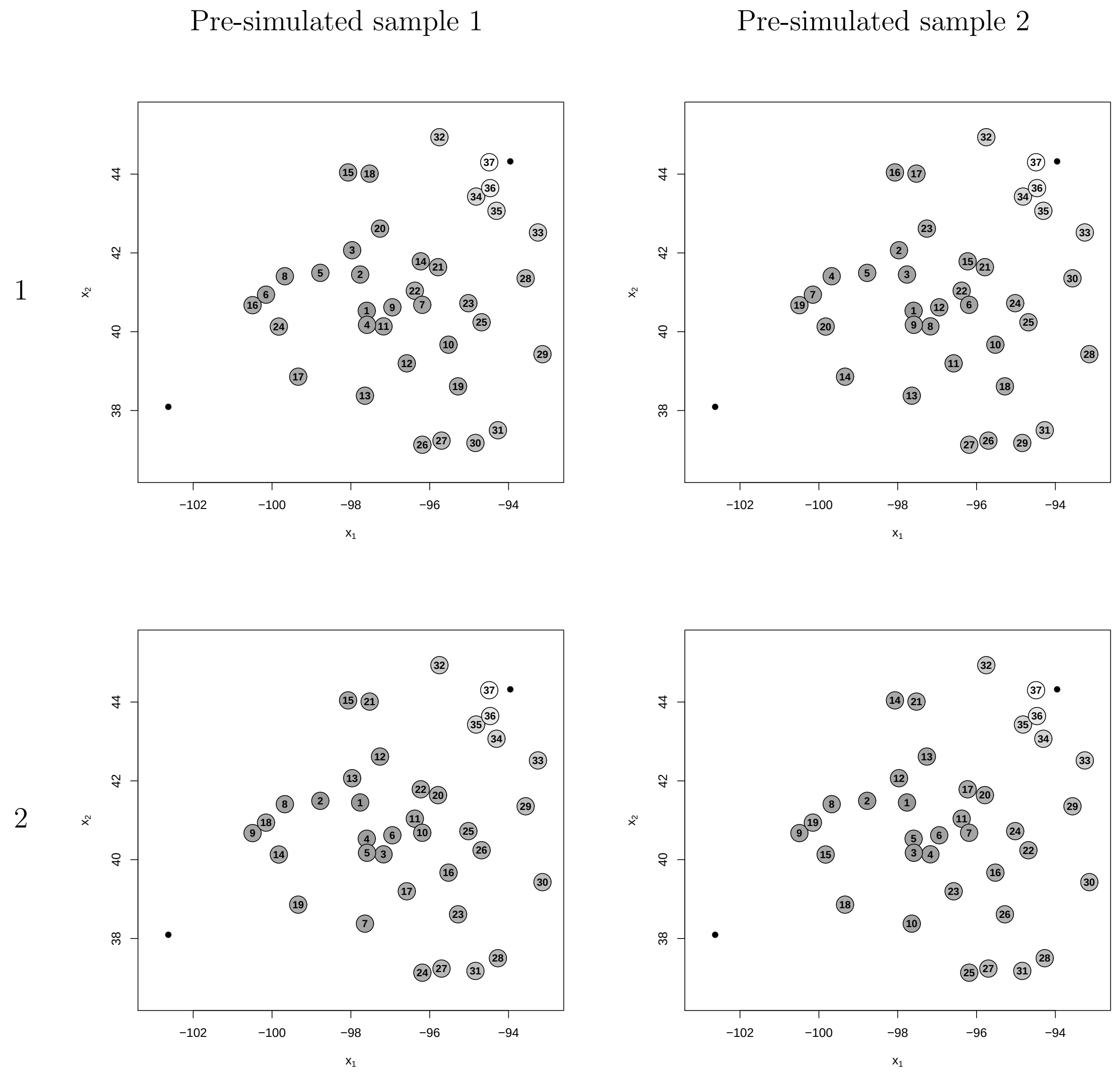

Figure 3: Various simulation runs showing the rankings of the expected utility criterion $\hat{U}(\boldsymbol{\xi}) = K^{-1}\sum_{k=1}^{K} \hat{u}_{LF}(\mathbf{z}^{(k)}, \boldsymbol{\xi})$, where $\hat{u}_{LF}(\mathbf{z}^{(k)}, \boldsymbol{\xi}) = 1/\widehat{\mathrm{Var}}_{\boldsymbol{\xi}}(\lambda|\mathbf{z}^{(k)})$ is the ABC posterior precision utility of the range parameter, when using the importance weight update method with the ABC posterior in Section 5.2 of the main article as input prior:
the **columns** depict the results for the two different pre-simulated samples, the **rows** depict the results for the first and second run of the Monte Carlo integration scheme

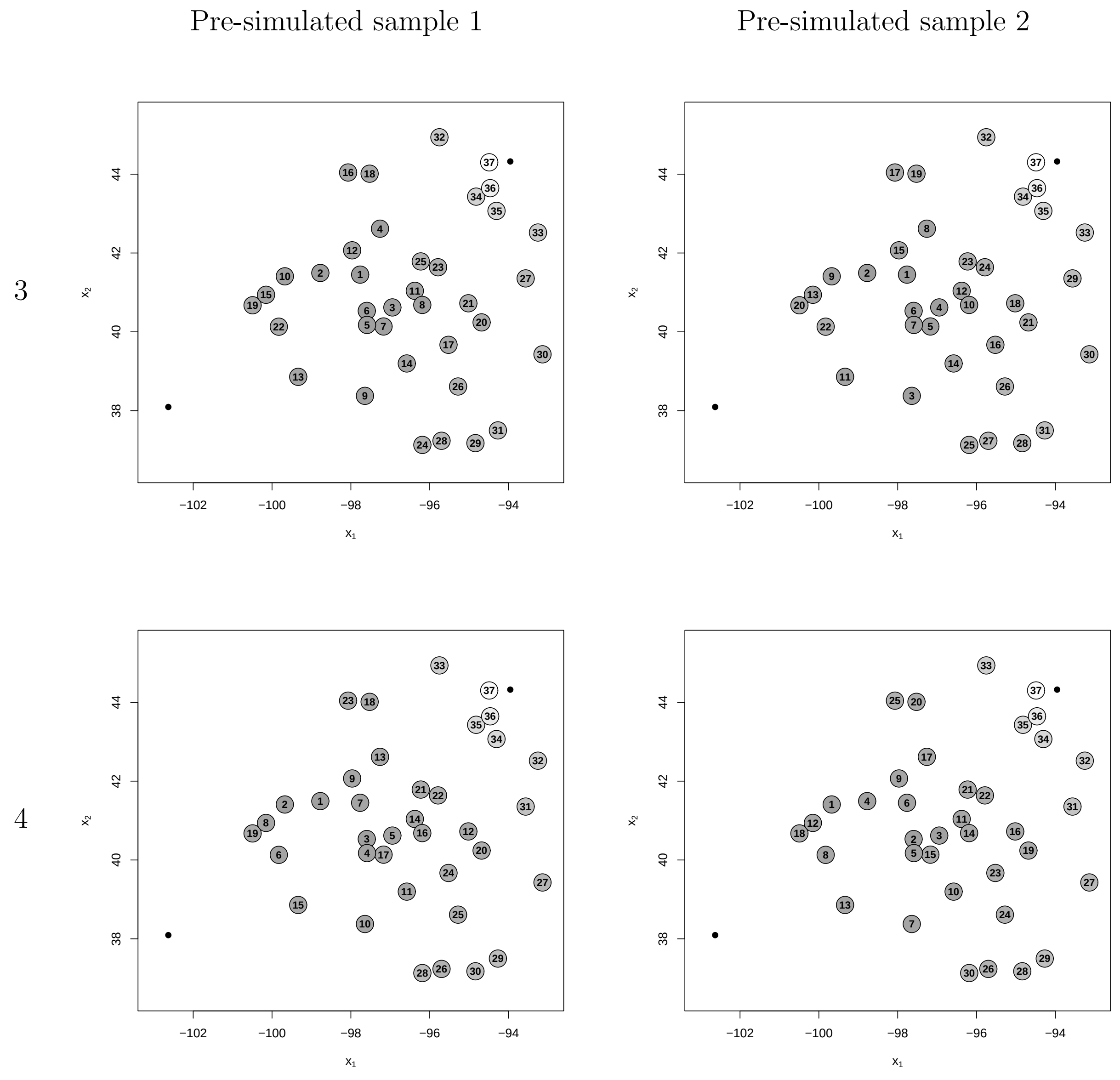

Figure 4: Various simulation runs showing the rankings of the expected utility criterion $\hat{U}(\boldsymbol{\xi}) = K^{-1}\sum_{k=1}^{K} \hat{u}_{LF}(\mathbf{z}^{(k)}, \boldsymbol{\xi})$, where $\hat{u}_{LF}(\mathbf{z}^{(k)}, \boldsymbol{\xi}) = 1/\widehat{\mathrm{Var}}_{\boldsymbol{\xi}}(\lambda|\mathbf{z}^{(k)})$ is the ABC posterior precision utility of the range parameter, when using the importance weight update method with the ABC posterior in Section 5.2 of the main article as input prior:
the **columns** depict the results for the two different pre-simulated samples, the **rows** depict the results for the third and fourth run of the Monte Carlo integration scheme
